# Simulating the Helicase Enzymatic Action on ds-DNA: A First-Principles Molecular Dynamics Study

**DOI:** 10.1021/acsomega.4c08555

**Published:** 2025-01-17

**Authors:** Angel Ivan Rodriguez-Leon, Cristian Ordóñez, Ruben Santamaria

**Affiliations:** † Department of Theoretical Physics, Institute of Physics, Universidad Nacional Autónoma de México, Ciudad de México 04510, Mexico; ‡ Department of Condensed Matter, Universidad Nacional Autónoma de Honduras, Tegucigalpa 11101, Honduras

## Abstract

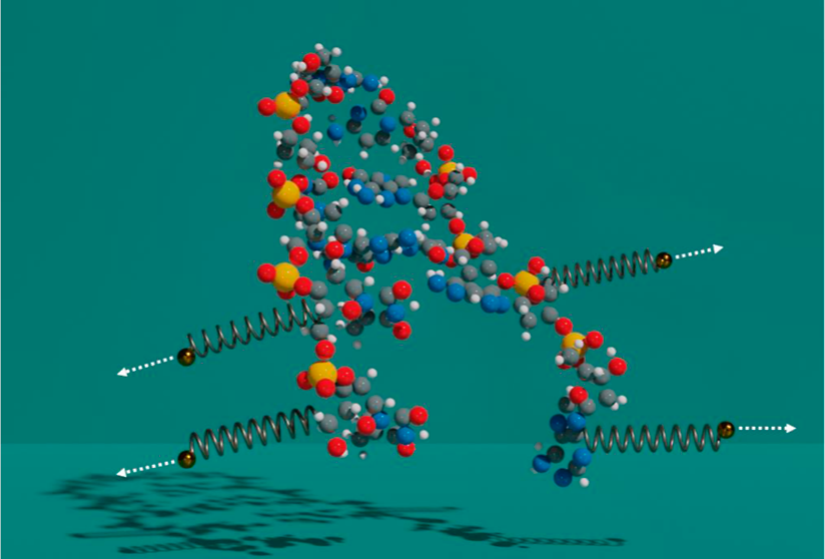

Understanding *DNA* replication is fundamental for advancements in fields such as genetics, molecular biology, and medical research. In this study, we investigate the mechanical characteristics of three distinct double-stranded *DNA* molecules (*ds-DNA*) as each of them is unwound into two individual single strands. To simulate the helicase action, the double strands are subjected to Langevin forces. By use of sequential and helical steering harmonic forces that simulate the enzymatic action of a helicase, each strand of *ds-DNA* is opened. The research focuses on determining thermal fluctuations, energy changes, charge variations, and individual forces associated with the separation of each base pair in the examined sequences. The findings emphasize the importance of combining quantum mechanical techniques with an implicit force model. This integrative approach is versatile and provides valuable insights into the essential processes governing *DNA* mechanisms, particularly in relation to cellular functioning, thereby enhancing our understanding of biological molecules.

## 1 Introduction

*DNA* exhibits mechanical properties that make it unique in diverse cellular processes. It possesses the strength to safeguard genetic information under ambient conditions while maintaining the structural flexibility for cellular replication, transcription, and translation.^1−3^ These processes are possible due to noncovalent forces between base pairs, which are composed of hydrogen bridges and stacking interactions.^1,4,5^ In nucleic acids, a hydrogen bridge forms between a donor group (X–H, where X is typically nitrogen or oxygen) and an acceptor group (Y–Z, where Y is an electronegative atom, such as nitrogen or oxygen). This interaction creates a noncovalent bond represented as X–H···Y–Z. In adenine, A, and thymine, T, hydrogen bridges involve N–H groups of adenine interacting with the oxygen atoms of thymine. Similarly, in guanine, G, and cytosine, C, complementary N–H and oxygen groups form hydrogen bridges.^6^

The dissociation of the double helix requires the action of enzymes to accelerate such a process. In particular, a helicase moves sequentially along the *DNA* double helix to promote strand separation using energy from the ATP hydrolysis.^7,8^ The study of the helicase enzymatic behavior, as well as the mechanical properties of the *DNA* under applied forces, is important to understand the way nature works at the nanoscale level, with the possibility to apply such knowledge in nanotechnology.^9^

Several theoretical and experimental investigations have been performed to analyze the mechanical response of force-induced separation of *ds-DNA*.^10−12^ Single-molecule manipulation experiments have allowed for the study of *DNA* destabilization with high resolution. For example, experimental techniques based on the use of an atomic force microscope (AFM) and optical and magnetic tweezers have been utilized to adhere one end of the biomolecule to a surface, while the other end is manipulated with a sensor to measure the applied force.^13^ In particular, magnetic tweezers have been favored to study the unzipping of *ds-DNA* strands under constant force and the stretching along the helical axis to establish the shear force.^14^ Also, optical tweezers combined with *DNA* origami have been applied to characterize the stacking forces without disrupting hydrogen bridges.^15^

Molecular simulations have been used as tools to complement experimental investigations. Classical molecular dynamics is convenient for analyzing biological systems at the atomic level.^16^ The separation of *ds-DNA* has been investigated using two distinct methods: stretching the molecules along the helical axis, commonly known as pulling,^13,17−20^ and perpendicular to the helical axis, known as unzipping.^21−24^ These methods are designed to analyze the forces contributing to the dissociation process and to understand the flexible *DNA* properties.

In a study by Naserian-Nik et al., the mechanical behavior of *DNA* was examined by employing a dummy atom and a virtual spring attached to one end of the structure. The focus was on studying the pulling-angle dependencies of the separation forces along the helical axis. The results highlighted the effectiveness of implicit solvent models in replicating the stretching process of *ds-DNA*.^13^ Santosh and Maiti delved into temperature-dependent forces by conducting *DNA* openings under both pulling and unzipping processes. Their observations revealed that the critical force of unzipping decreases with an increase in temperature, and distinct force-value jumps were identified due to variations in the nucleic acid sequence.^21^*DNA* with intercalated drugs using various pulling methods has been investigated by Sahoo et al. Their study compared dissociation forces between bare *ds-DNA* molecules and *ds-DNA/drug* complexes. The findings indicated a reduction in forces for the latter and also suggested that intercalators impede the dissociation process.^23^

These investigations shed light on the diverse dependencies of *DNA* dissociation forces, enhancing our understanding of the *ds-DNA* opening processes. It is noteworthy, however, that classical molecular dynamics heavily relies on the chosen force field and often fails to incorporate relevant quantum mechanical effects crucial for analyzing hydrogen bridge breakage.

A steering first-principles molecular dynamics simulation for dissociation of the nucleic acid bases was investigated by Ordóñez et al. They demonstrated the importance of the solvent environment and evaluated the forces of hydrogen bridges during the dynamic rupture of the base pairs with harmonic external forces, avoiding out-of-plane torsional effects of the AT and GC nucleic acid bases.^25^ Atomistic models provide detailed insight into the stability of *DNA*. Recent progress in high-performance computing enables the incorporation of both the *DNA* backbone and the top-and-bottom adjacent base pairs in these models. These inclusions are particularly significant as they provide information about the stacking interactions that play a crucial role in maintaining the stability of the double helix.^26^

To our knowledge, there are no existing investigations employing a separation protocol resembling the helicase dissociation process of *DNA*. Helicases break hydrogen bridges in a helicoidal-sequence manner along the nucleic acid chain. In this context, first-principles molecular dynamics studies that replicate biological conditions and apply helicase-type forces are helpful in characterizing the enzymatic action of helicases on *ds-DNA*.

The main purpose of this research is to investigate the dissociation of short *ds-DNA* sequences using steered molecular dynamics simulations in conjunction with the Langevin force approach. Within this framework, temperature and friction are considered dynamic and static interaction modes between the helicase and nucleic acid bases. This enables us to capture the essential aspects of helicase–*DNA* interactions. Our separation process between strands simulates the helicoidal-sequential manner in which the helicase, implicitly considered, acts on *DNA*. To analyze possible sequence dependencies, we change nucleic acid bases in the strands and evaluate forces, energies, charges, and thermal fluctuations with and without dispersion corrections.

This work is organized as follows: the molecular dynamics method and the protocol on the *DNA*-strand separation (including the sequential action of the harmonic forces and the Langevin force approach) are presented in the next sections. The last modules give the main findings of this study.

## 2 Methods

In this research paper, we employ a quantum mechanical treatment for the electronic structure calculations and a classical approach for nuclear motion, all operating under the Born–Oppenheimer approximation.

Density functional theory (DFT) is chosen for solving the time-independent Schrödinger equation due to the computational efficiency and relatively accurate results of this method.^27−29^ In particular, DFT has garnered increasing attention within the biological field due to its favorable results.^10,30−32^
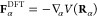
1a
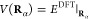
1b

Utilizing DFT eliminates the need to deal with model potentials and force fields to simulate atomic interactions. The electronic energy, *E*^DFT^, calculated by DFT serves as the potential energy in which the nuclei are immersed. The force acting on atom α is **F**_α_^DFT^, which is calculated from the potential *V*(**R**_α_). Accurate DFT calculations rely on selecting an appropriate exchange–correlation functional for the system under investigation. In this study, we employ the B3LYP/6-31g* and D3 dispersion correction term level of theory, which has been shown to produce adequate results for nucleic acid systems.^33−35^ Many of the existing proposals for dispersion corrections are empirical in nature. These corrections can either positively or negatively affect energy descriptions as the effects of dispersion are dependent not only on the magnitudes of the molecular dipoles but also on their orientations. Furthermore, the quantification of dispersion interactions is still a topic of ongoing debate, and there is currently no definitive method to accurately assess the efficacy of these dispersion proposals in energy calculations.^36−38^ Recognizing this, we have opted to also present results that include no dispersion forces, thereby acknowledging the ongoing debate surrounding dispersion interaction quantification.^39,40^

The computations were conducted using two programs: (1) the commercially available *TeraChem* software^41^ and (2) our proprietary in-house code *Interactive Molecular Dynamics* (*IMD*). The *IMD* code performs molecular dynamics simulation and includes forces calculated on the fly by *TeraChem*. The code will be officially released to the community in a separate publication. Energy changes, Mulliken charges, and molecular forces are computed based on the DFT-D3 level of theory aforementioned.

### 2.1 Molecular Model

The intricate interplay between the *DNA* helix, helicase, and the surrounding aqueous solvent environment is crucial in understanding the opening of a double *DNA* helix. However, simulating the helicase–*DNA* interaction under such realistic conditions poses significant computational challenges. To address this, we employ a force model, namely, the Langevin approach. This method allows us to account for the static and dynamic interactions between the helicase and *DNA* by introducing friction and kicking terms, statistically capturing the essential aspects of helicase–*DNA* interactions. While this approach may sacrifice some degree of realism, it allows us to perform the necessary calculations and gain valuable insights into the helicase–*DNA* system. The sensitivity of the *DNA* unzipping process to temperature variations remarks the importance of incorporating temperature and friction effects in our simulations.^21,42^

The charged structural portion of *DNA* consists of phosphate groups situated on the external side of the molecule, while the internal region is composed of neutral nucleic bases. This internal region is occupied by not only the *DNA* molecule itself but also the helicase during biological processes, preventing the presence of water molecules. Consequently, distance waters and their electrostatic interactions with the *DNA* segments play a secondary role in the analysis of hydrogen bridge breaking. The equation of motion of the *DNA* atom with label α is
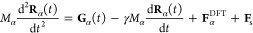
2

The thermal agitation is provided by the stochastic term, **G**_α_. The strength and direction of this force are obtained from a bivariate distribution function in the particle position and velocity. The distribution function contains the macroscopic features of the environment such as the viscosity and temperature. Detailed information on this distribution function, originally determined by Chandrasekhar et al., is found in refs (43–46). The second term describes the viscous drag force. To reflect the environmental conditions of the helicase–*DNA* interaction, we selected a friction coefficient slightly higher than that typically used for systems in water alone, namely, γ = 4.0 ps^–1^. This accounts for the additional mechanical friction imposed by the helicase as it interacts with and holds the DNA during the unwinding process. While this choice represents a reasonable starting point, we acknowledge that determining an optimal value is challenging due to the vastly different time scales of experiments and simulations, making empirical tuning necessary. The last two terms represent the systematic forces, which correspond to the ground-state electronic energy calculated with DFT, eq 1a, and the external force, **F**_s_, applied to the *DNA* molecule to achieve strand separation. This last force is applied only to specific atoms of the *DNA* molecule (to be discussed later).

The energy exchange between the *DNA* molecule and the implicit helicase environment is accounted for by the friction drag force and the stochastic term. The temperature used in the simulation is 300 *K* (≈27 °C). Due to the statistical nature of the Langevin terms, thermodynamic equilibrium is achieved when the mechanical temperature derived from the *DNA* atoms corresponds to the statistical temperature imposed by the distribution function. The Langevin equation is solved using a Langevin integrator, which is similar to a Verlet numerical integrator.^47,48^ The time step in the simulations is 1 fs, corresponding to the vibrational period of hydrogen (the lightest element in the molecular system) and divided by a factor of 10 to ensure an acceptable resolution time for the simulation.

This methodology allows us to conduct quantum mechanical calculations, offering a notable reduction in computational expenses. Various studies, including our own previous works, as well as those by other researchers, support the effectiveness of this approximation.^13,17,23,25^ Although this offers advantages, one notable drawback is that it does not explicitly account for the occupation of the internal region by helicase during biological processes. Despite this limitation, the Langevin force approach successfully integrates the pertinent effects of helicase in an implicit manner during the hydrogen bridge rupture process.

### 2.2 Simulation of Helicase Enzymatic Action

**Figure 1:**
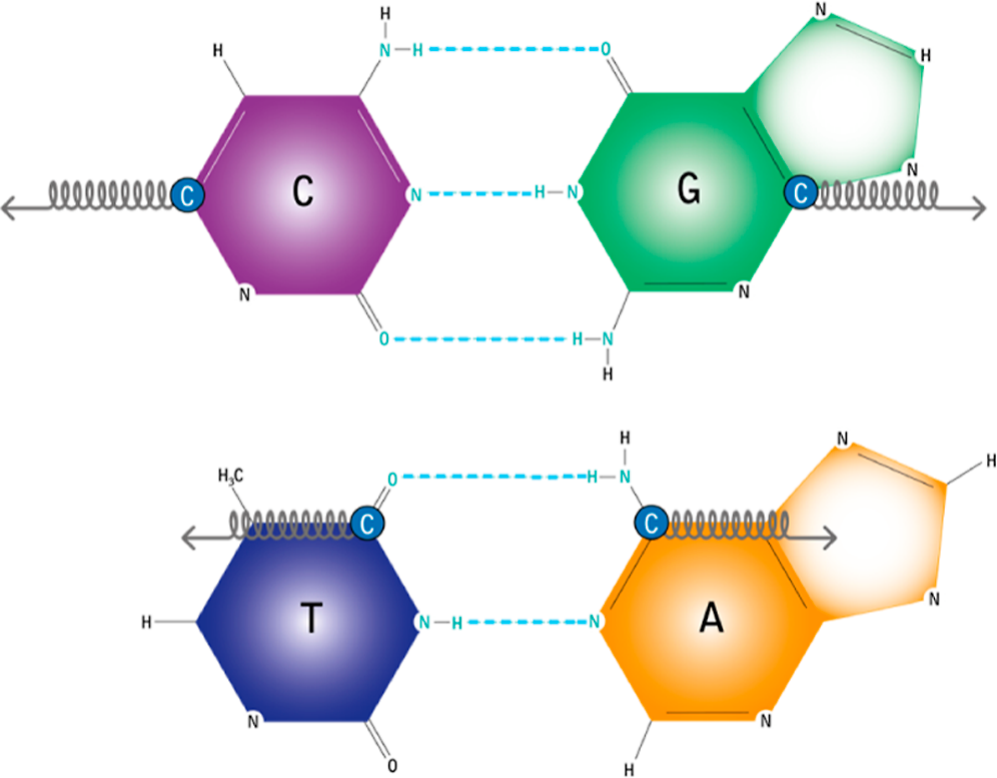
Positioning of springs on each base pair.

The present study outlines the development of a model for helix dissociation via molecular dynamics. It is based on the use of springs, where they are attached to specific atoms of the conjugated nucleotides. The springs are moved in a sequential manner and positioned in the helicoidal form around the *DNA* double helix to simulate the enzymatic action of a helicase in the cellular processes. The chosen atoms—C4 of thymine, C6 of adenine, C5 of cytosine, and C5 of guanine—define a line of action along the hydrogen bridges. This line changes based on the specific base pair (AT or GC) under examination. Its purpose is to minimize unwanted torsional moments during the separation of Watson–Crick base pairs. The molecular setup is depicted in Figure 1. The steering model based on harmonic forces has been successfully employed in similar studies involving nucleic acid bases.^25^

The spring stiffness is *k* = 0.01 N/cm. The springs are elongated with one of the tips of the spring moving with constant velocity, **v** = 0.002 Å/fs. Such values ensure a smooth opening of *ds-DNA* under quasi-equilibrium conditions. Spring stiffness minimally affects *DNA* dissociation force calculations, especially at low pulling speeds. Experiments typically report pulling rates, instead of spring stiffness. Despite differences in techniques such as AFM and optical/magnetic tweezers, which lack helicoidal breaking, the overall rupture force behavior remains consistent across diverse experiments and simulations.

The equation of the spring force acting on a selected carbon atom of a base pair is
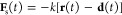
3

The instantaneous position of the carbon atom is **r**(*t*), and **d**(*t*) = **r**(*t*) – **v***t* is the distance between the current position of the atom and the spring pulling tip. The spring force increases linearly with time. If we define the force per unit time as the pulling rate, then the product *k***v** in our case is 0.20 pN f s^–1^.

The instantaneous potential energy of the carbon atom due to the spring force is given by
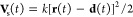
4

This potential generates the force **F**_s_ of eq 2. The spring acting on the selected carbon atom of the complementary nucleic acid base demands an opposite sign in the expression **d**(*t*) = **r**(*t*) + **v***t* (Figure 1). The two forces that we calculate on every base pair resemble the forces that two people exert by pulling a rope on opposite ends. In this respect, we report one of these two forces because it corresponds to the “tension exerted on the rope”. The action of the springs ultimately results in separation of the particular base pair under examination. The nucleic acid bases forming the base pair are considered to be separated when the distance between their centers of mass (CM) reaches a value of 8.2 Å.^17,49^ This is a general threshold applied to all of the separated nucleic acid bases. The average separation distance between the CM of G and C and A and T is 5.8 Å when the two strands are equilibrated and linked to each other.

Once the first base pair is spatially separated, the spring forces are switched off, and a new pair of spring forces acting on the second base pair is switched on. The new spring forces define new action lines which are rotated with respect to the old ones since the *DNA* molecule is shaped like a twisted ladder. The spring forces taken in this way unwind *DNA* without entanglement. Once the bases of the second base pair are separated, the process is repeated sequentially, following the helicity of the remaining *DNA* base pairs. The sequential action of the spring forces results in the separation of *ds-DNA* into two *ss-DNA*.

**Figure 2:**
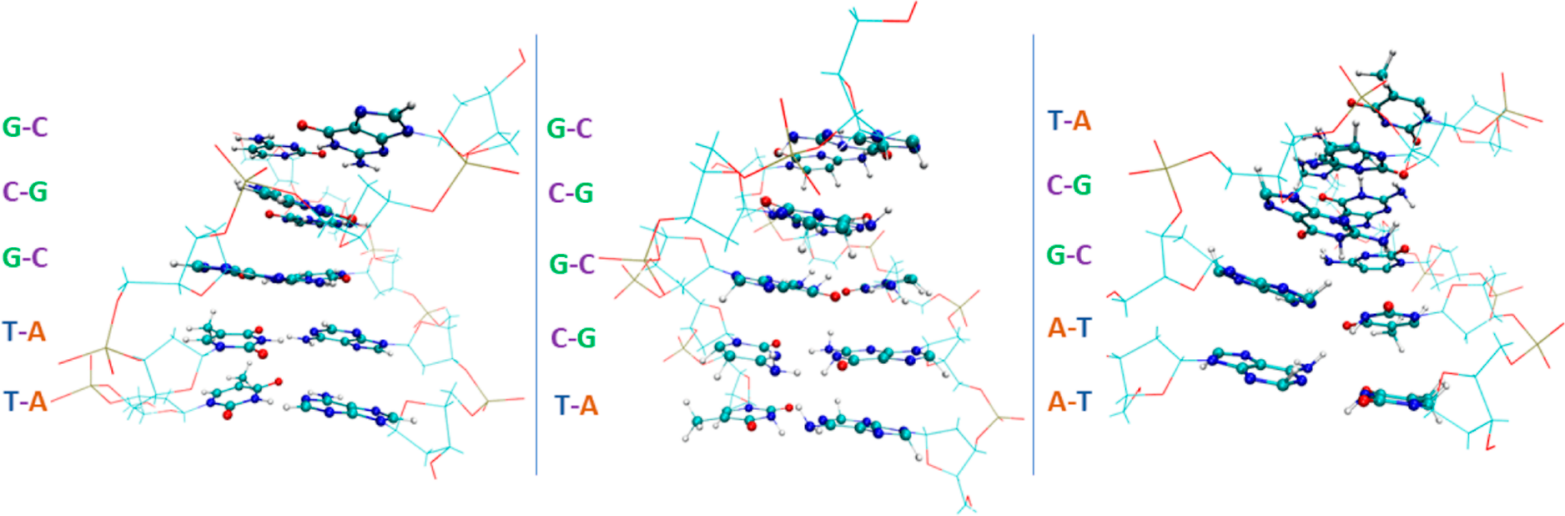
*DNA* sequences with their specific nucleic acid bases.

Due to the high computational demand that first-principles simulations require, we focus our attention on short *ds-DNA* structures composed of five base pairs. The separation of nucleic acid base pairs is not influenced by nucleic acid bases located far away from these under study.^22^ Our study analyzes the following three short sequences (Figure 2):

- T–T–G–C–G
- T–C–G–C–G
- A–A–G–C–T

Our objective is to examine heterosequences with biological relevance to evaluate the impact of sequence variations on dissociation forces. Specifically, the chosen sequences exhibit a progressive increase in the number of GC base pairs, ranging from two to four, while keeping the central base pair constant (of GC type). The central GC base pair serves as a common reference point across the three sequences, facilitating value comparisons. The initial structures of the short *DNA* helix segments are not critical to the simulations, as such segments undergo conformational changes due to thermalization throughout the dynamics.

## 3 Results and Discussion

According to the previous discussion, it is possible to characterize the dissociation of the nucleic acid base pairs using harmonic forces. Our work differs from other studies in that we take into account small sequences of *DNA* including sugars and phosphates, as well as the stacking interactions that occur in *DNA* dissociation. The quantum mechanical treatment allows one to report charge transfers, energy variations, and thermal fluctuations during the double strand separation. One additional goal is to establish force variations with respect to the nucleic acid sequences.

The simulation of each *DNA* structure is performed in three stages:

1. The *DNA* sequences are energetically equilibrated under no external forces.
2. The dissociation process is started with the action of spring forces and finishes when the threshold separation distance between all base pairs of the *DNA* sequence is achieved.
3. The two *DNA* strands are completely separated and equilibrated without the action of the external spring forces.

The temperature and energy fluctuations during the three stages are discussed and illustrated for all *DNA* sequences in the following sections. Additionally, calculations both with and without dispersion corrections are presented to evaluate the importance of incorporating these corrections in the analysis of the *DNA* dissociation forces.

### 3.1 Temperature and Energy Fluctuations

A key strength of molecular dynamics lies in its ability to capture time-dependent processes, offering statistical insights into the energies, charges, thermal equilibration, and other critical properties. Since *DNA* helix opening is inherently dynamic, molecular dynamics is invaluable for uncovering the details of this process. To fully understand its time-dependent behavior, it is essential to present plots that highlight the evolving characteristics of the system, illustrating each stage of *DNA* separation.

The simulation begins by equilibrating the system to a temperature of 300 K, during which the *DNA* strands remain linked. Once the structures are in their equilibrium state, the dissociation protocol is started. In this part of the simulation, the springs take action on the base pairs and give energy to the system. After an interval of time, which depends on the *DNA* sequence, the base pairs are separated.

**Figure 3:**
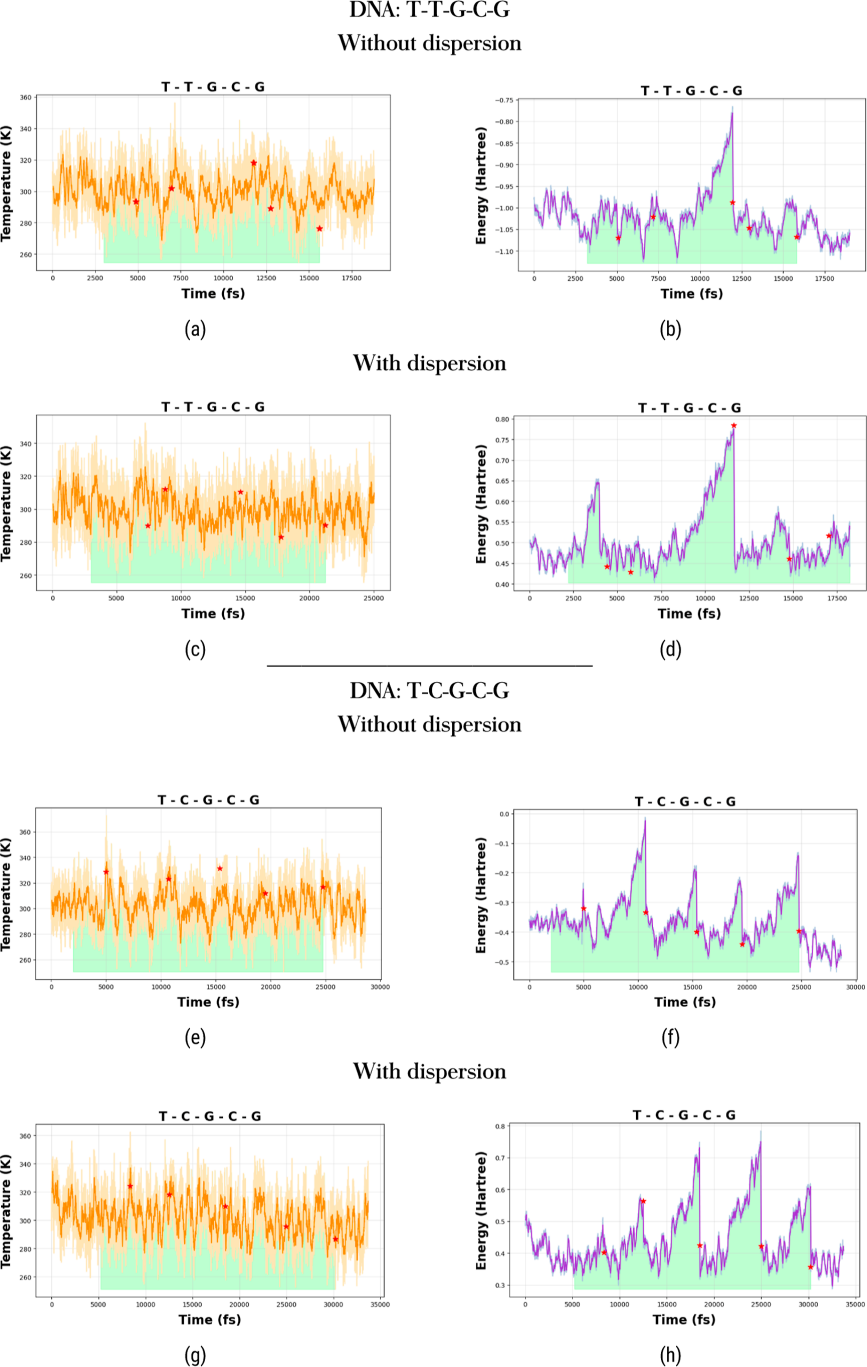
Temperature fluctuations are shown in the left panels (top: without dispersion; bottom: with dispersion), and energy fluctuations are depicted in the right panels (top: without dispersion; bottom: with dispersion) for the T–T–G–C–G (a–d) and T–C–G–C–G sequences (e–h). Asterisks mark the instances of base pair separations. Shaded regions highlight the time intervals during which harmonic forces are applied to the nucleic acid bases.

**Figure 4:**
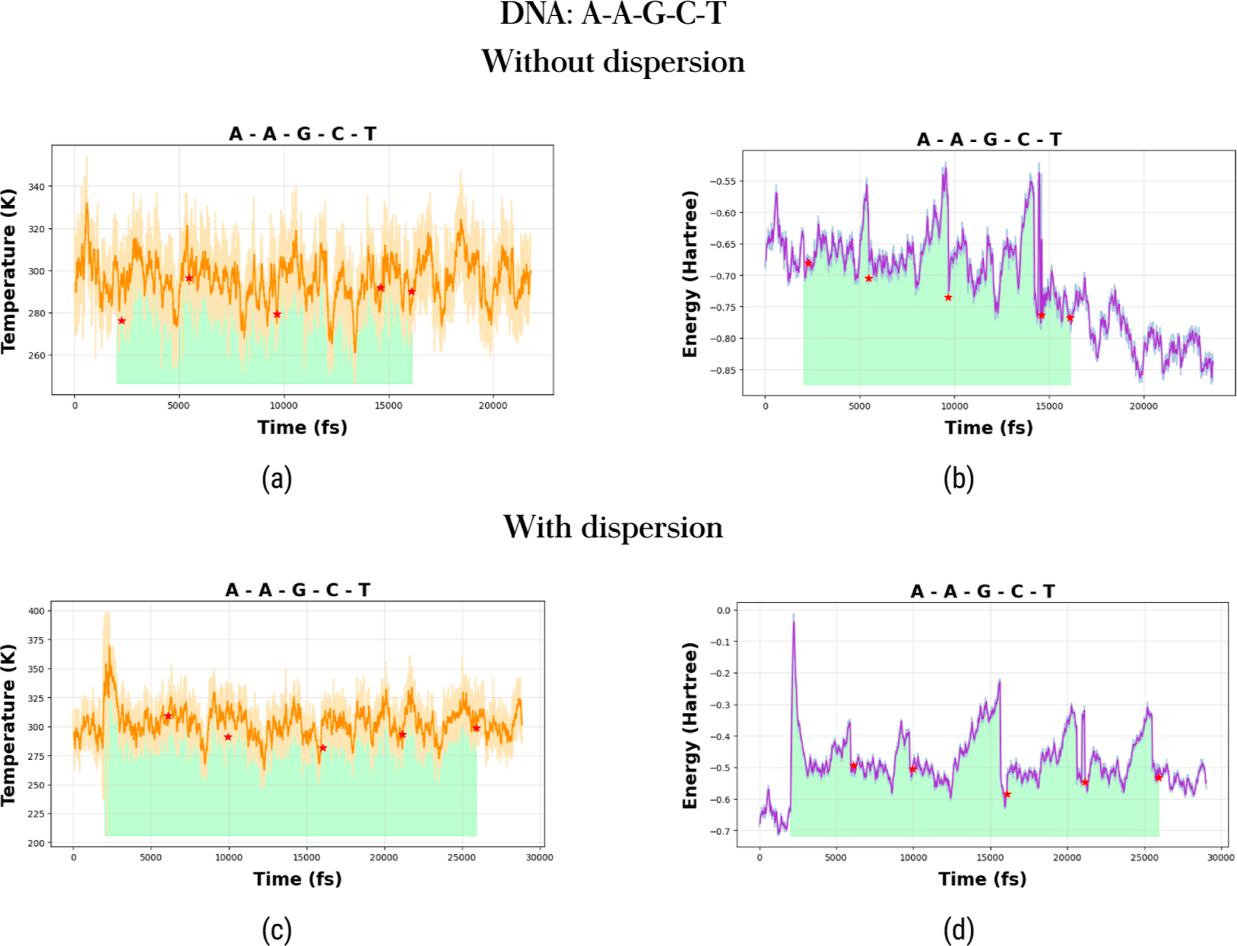
Temperature fluctuations are shown in the left panels (top: without dispersion; bottom: with dispersion), and energy fluctuations are depicted in the right panels (top: without dispersion; bottom: with dispersion) for the A–A–G–C–T sequence (a–d). Asterisks mark the instances of base pair separations. Shaded regions highlight the time intervals during which harmonic forces are applied to the nucleic acid bases.

From Figures 3 and 4, we observe that the temperature remains relatively unaffected by base-pair separations. However, the energy is highly sensitive to these separations. This behavior is consistent for calculations with and without dispersion corrections.

### 3.2 Dissociation Forces

**Figure 5:**
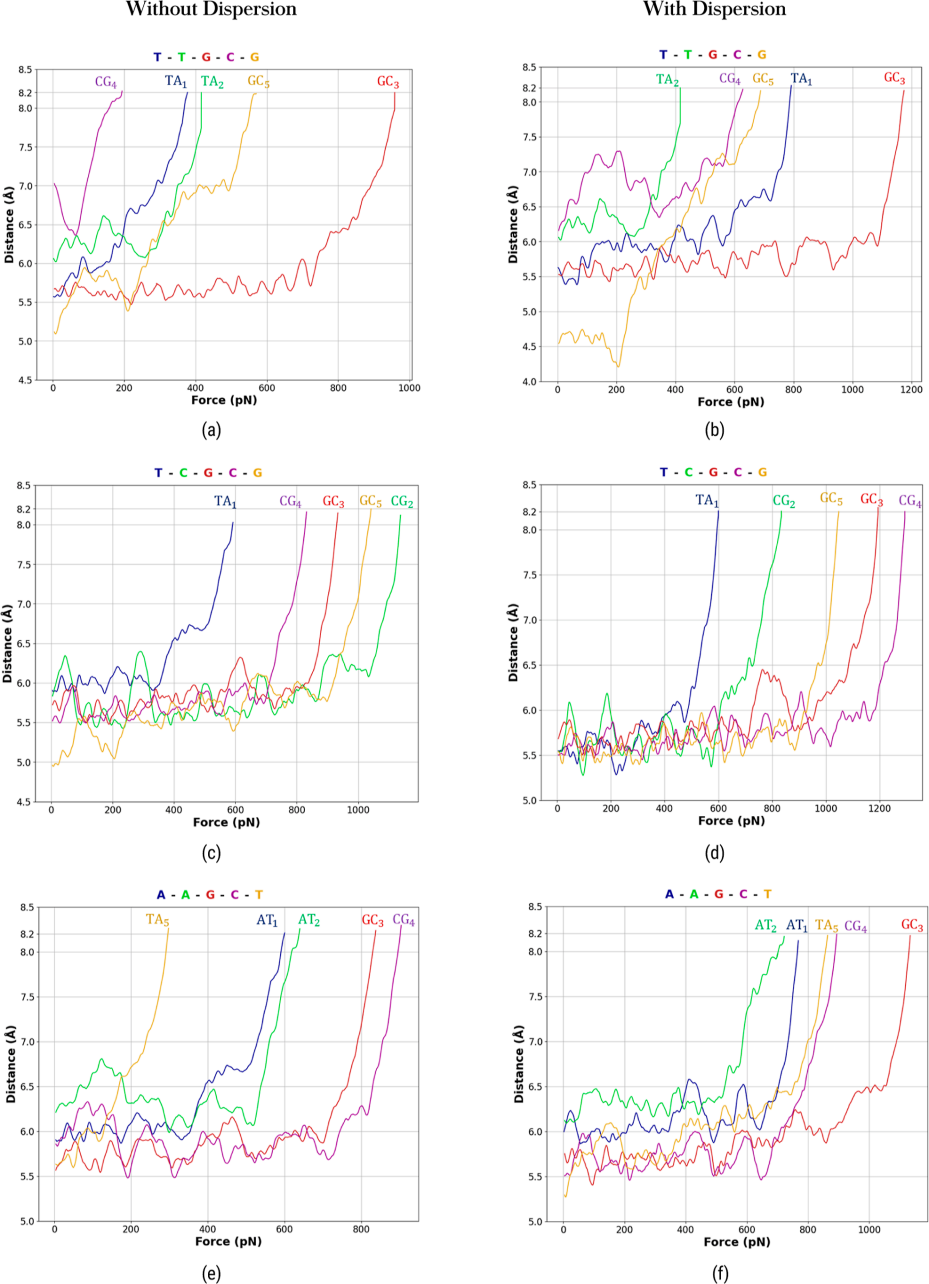
CM separation vs dissociation force for each sequence: T–T–G–C–G (a,b), T–C–G–C–G (c,d), and A–A–G–C–T (e,f). The left-hand side shows data without dispersion, while the right-hand side includes dispersion. CM separation refers to the distance between the CM for adenine and thymine and similarly for cytosine and guanine.

Every single base pair may be considered as a ductile compound susceptible to rupture under the action of external forces. In the present case, the applied springs gradually increase the force on each base pair, until the hydrogen bridges are gently broken. There are two important moments that characterize the dissociation forces. The first instant is featured by a force that produces a relatively rapid separation between each base pair (Figure 5). Such a force is called the rupture force, *F*_rup_. The second instant is characterized by a force that reaches the threshold separation of 8.2 Å (previously discussed) between each base pair. It is called maximum force, *F*_max_. Note that *F*_max_ is an upper bound to *F*_rup_ because a base pair appears to be separated before reaching *F*_max_. However, the value of *F*_rup_ cannot be accurately assigned an exact value, because the breaking of a base pair is not an abrupt observable phenomenon. In spite of that, we have computed for practical purposes an *F*_rup_ value for each base pair. It is established by the point where the distance between the mass centers of the bases forming the pair ceases to decrease. Once the *F*_rup_ force is attained, we witness a swift separation of the nucleic base pair, indicating that the externally applied harmonic forces successfully overcome the hydrogen bridge strengths. The magnitude of the *F*_rup_ depends on the specific base pair and its position in the strand. Table 1 summarizes the *F*_rup_ and *F*_max_ values of the three analyzed *DNA* structures with and without dispersion corrections.

**Table 1:** Dissociation Forces of Base Pairs^a^

nucleic sequence	*F*_rup_ (pN)	*F*_max_ (pN)	*F*_teo_^b^ (*F*_exp_^c^) (pN)
T–T–G–C–G			
1 bp (T–A)	307.972 (**723.958**)	377.509 (**792.011**)	630 (550)
2 bp (T–A)	387.972 (**390.00**)	416.067 (**420.036**)	630 (550)
3 bp (G–C)	922.022 (**1113.990**)	958.274 (**1173.970**)	1080 (860)
4 bp (C–G)	65.994 (**561.979**)	194.027 (**627.974**)	1080 (860)
5 bp (G–C)	511.969 (**489.971**)	569.971 (**688.036**)	1080 (860)
T–C–G–C–G			
1 bp (T–A)	519.029 (**514.035**)	591.969 (**600.043**)	630 (550)
2 bp (C–G)	1107.976 (**732.032**)	1137.966 (**834.112**)	1080 (860)
3 bp (G–C)	887.995 (**1123.959**)	933.969 (**1193.991**)	1080 (860)
4 bp (C–G)	721.980 (**1248.038**)	831.970 (**1294.012**)	1080 (860)
5 bp (G–C)	950.035 (**1004.000**)	1041.982 (**1047.996**)	1080 (860)
A–A–G–C–T			
1 bp (A–T)	536.027 (**721.980**)	600.538 (**768.036**)	630 (550)
2 bp (A–T)	575.986 (**577.963**)	640.002 (**721.980**)	630 (550)
3 bp (G–C)	781.960 (**1055.988**)	837.985 (**1134.011**)	1080 (860)
4 bp (C–G)	764.020 (**864.020**)	903.979 (**897.010**)	1080 (860)
5 bp (T–A)	255.984 (**822.001**)	298.003 (**864.020**)	1080 (860)

a Dissociation forces with dispersion corrections are shown in boldface and within parentheses.

b Theoretical values obtained with AMBER 4.1 of a single nucleic base pair in solution.^50^

c Experimental values obtained from atomic force microscopy acted on a single nucleic base pair.^51^

The first examined sequence was T–T–G–C–G. The dissociation rupture force for the middle base pair, without and with dispersion corrections, has the highest value of the rupture force in this sequence. In contrast, the smallest rupture forces without and with dispersion corrections correspond to the 4CG and 2TA base pairs, respectively. In general, force values including dispersion corrections are higher than those without dispersion corrections except for the 5GC base pair. The overall force trends are as follows:

With dispersion corrections:

3*GC* > 1*TA* > 4*CG* > 5*GC* > 2*TA*.

Without dispersion corrections:

3*GC* > 5*GC* > 2*TA* > 1*TA* > 4*CG*.

The second sequence is T–C–G–C–G. It is characterized by a high content of GC base pairs, which are known for imparting not only elevated hydrogen bridging forces but also robust stacking interactions.^12^ The smallest value of the dissociation forces belongs to the first base pair, 1TA. This sequence is hard to break apart because it has high values of the rupture forces, mainly due to the strong bridge strengths of GC base pairs. The results provide valuable insights into the challenges faced by helicases when unwinding GC-rich sequences as opposed to those with a high content of AT base pairs. The overall force trends are as follows:

With dispersion corrections:

4*CG* > 3*GC* > 5*GC* > 2*CG* > 1*TA*.

Without dispersion corrections:

2*CG* > 5*GC* > 3*GC* > 4*CG* > 1*TA*.

The third sequence A–A–G–C–T has a high content of AT base pairs, whose rupture forces are in general lower than those of the second sequence with a high content of GC base pairs. The third base pair, 3GC, located at the middle of the sequence, presents the biggest resistance to opening. The overall force trends are

With dispersion corrections:

3*GC* > 4*CG* > 5*TA* > 1*AT* > 2*AT*.

Without dispersion corrections:

3*GC* > 4*CG* > 2*AT* > 1*TA* > 5*TA*.

**Figure 6:**
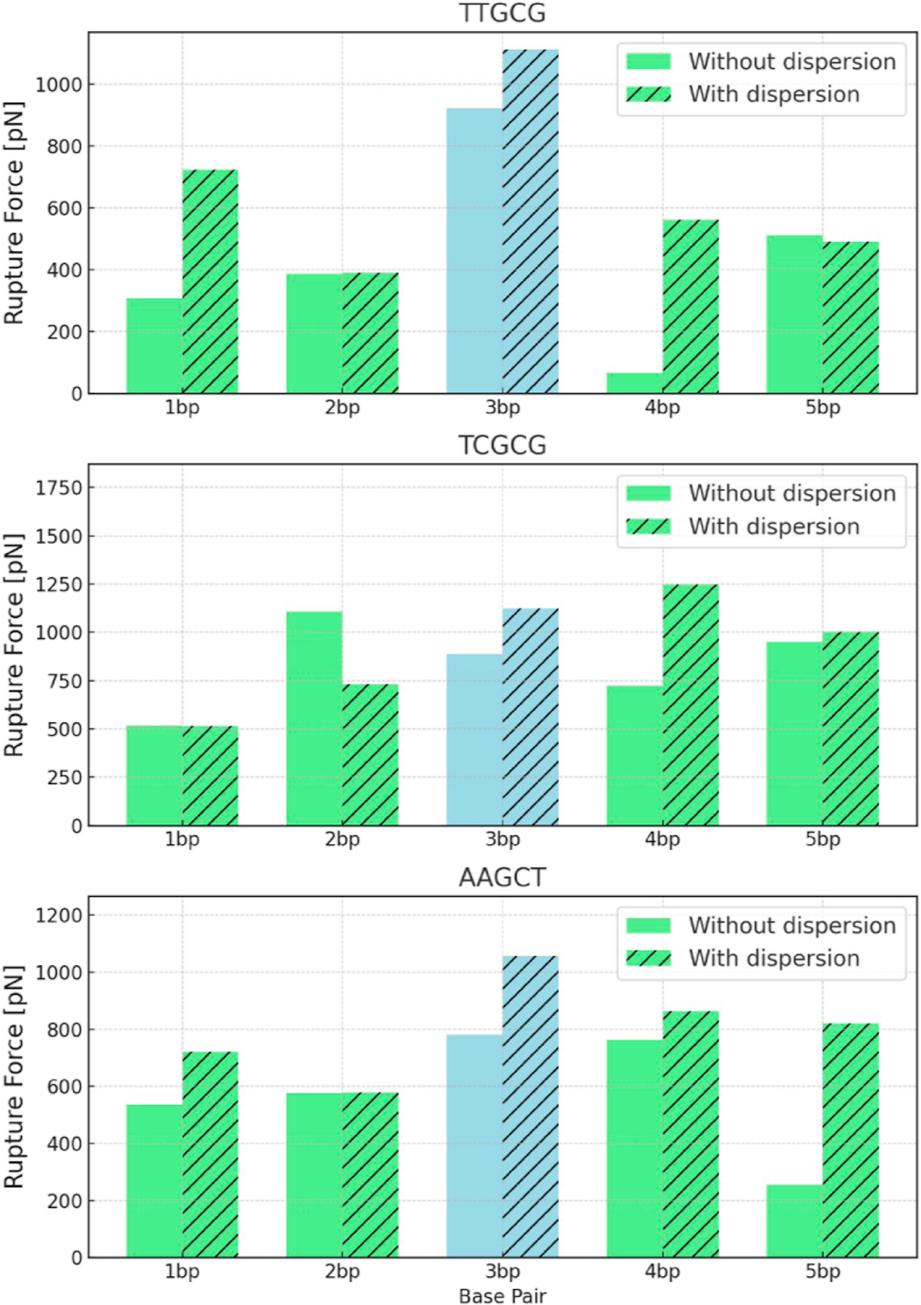
Magnitudes of the rupture forces with and without dispersion corrections for each base pair in the three *DNA* sequences analyzed in this work.

The analysis of the separation forces for the *DNA* sequences exhibits several important facts. (1) TA base pairs require relatively smaller forces than those associated with GC base pairs to be separated, and the magnitudes of the separation forces depend on the specific *DNA* sequence. (2) The separation forces of base pairs embedded in the *ds-DNA* are in general bigger than those of the base pairs located at the ends of the *ds-DNA* strand. The opening of a nucleic base pair has an influence on the behavior of neighbor base pairs, and this influence also depends on the type of nucleic base pairs, which makes it difficult to establish a general pattern. (3) Certain base pairs exhibit a considerable separation in the thermalization process and before the action of the springs (the Langevin force model integrates agitation effects in accordance with the predetermined temperature, capable of modifying the nucleic base separation). (4) Dispersion corrections typically lead to an increase in force values, and the extent of this effect varies across different *DNA* sequences as shown in Figure 6. Interestingly, the force values without dispersion corrections are shown to be lower with respect to those experimentally and theoretically reported in the literature. The values reported by other authors (last column of Table 1) correspond to isolated base pairs where the sugar–phosphate backbone was omitted. Finally, from the behavior of the forces in terms of distances (Figure 5), we observe an ample region where the dissociation forces show relatively small fluctuations, indicating resistance of the base pairs to separation. This behavior is followed by a rapid increase in force, leading to a rupture moment. Such a behavior is similar to that observed in models utilizing explicit water molecules,^13,17,21,23,25^ thus demonstrating that our model captures the key underlying mechanisms involved in the biological phenomenon.

### 3.3 Maximum Dissociation Forces

The maximum dissociation forces, *F*_max_, show behaviors similar to those of the rupture forces, as presented in Table 1. (1) *F*_max_ values with dispersion corrections (in bold face and in parentheses) are generally higher than those without dispersion corrections. The exceptions to this observation are 2*CG* of the T–C–G–C–G sequence and 4*CG* of the A–A–G–C–T sequence. (2) The magnitude of the forces varies significantly depending on the base pair sequence. (3) Generally, GC base pairs show higher rupture forces with respect to AT base pairs. This is consistent with the strong bonding presented by GC pairs due to their three hydrogen bridges compared to two hydrogen bridges of AT pairs. (4) The sequence context has influence on the individual rupture forces, as evidenced by force variations for the same base pair in different positions and sequences. (5) The dispersion correction seems to have a more pronounced effect on some base pairs than others, suggesting that the importance of dispersion forces may vary depending on the specific base pair and its context. (6) The 3*GC* base pairs in the sequences commonly show high forces with and without dispersion corrections.

### 3.4 Mulliken Electric Charges

**Figure 7:**
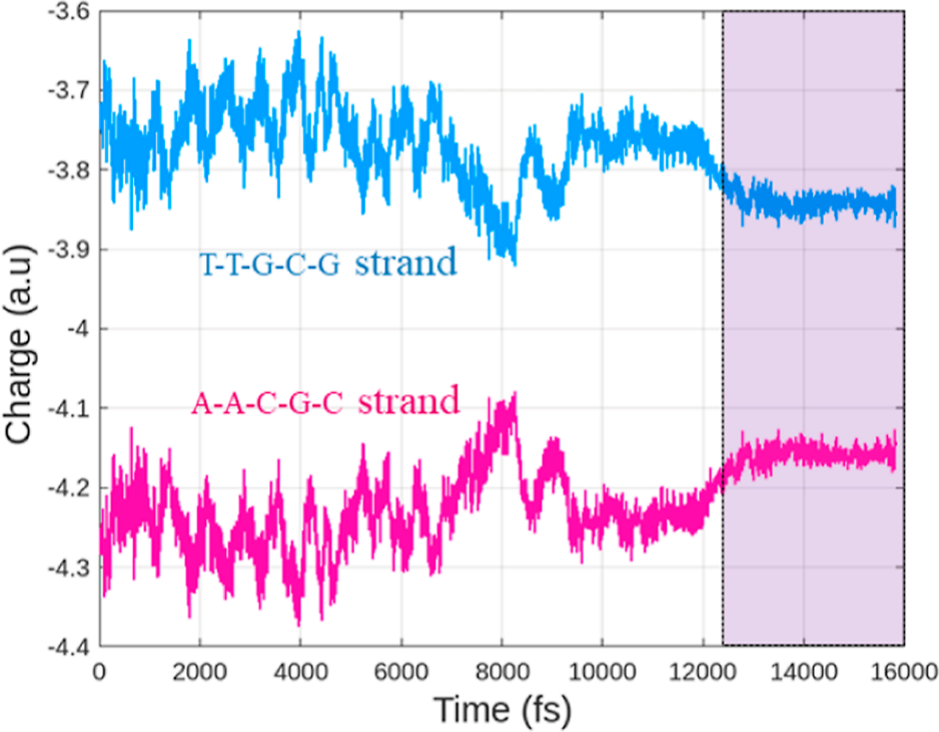
Mulliken charge variations of individual strands for the sequence T–T–G–C–G. The shadowed region indicates the time interval of total strand separation and equilibration.

In general, *DNA* is negatively charged due to the −1*e* charge associated with each phosphate group. This attribute is important for stability and molecular recognition.^52^ The electric charges for the analyzed *DNA* sequences in this work are computed following the Mulliken scheme. It is observed that all of the *DNA* molecules exhibit a similar pattern. As an example, the behavior in time of the electric charges for the first *DNA* sequence, T–T–G–C–G, is shown in Figure 7. During the double-strand dissociation, the harmonic forces induce oscillatory behavior in the atomic electric charges, leading to fluctuations. Once the strands are separated, the charges are stabilized, as shown in the shadowed region of Figure 7. The resulting product is two charged single nucleic strands, similar to those produced in cellular replication processes.

**Figure 8:**
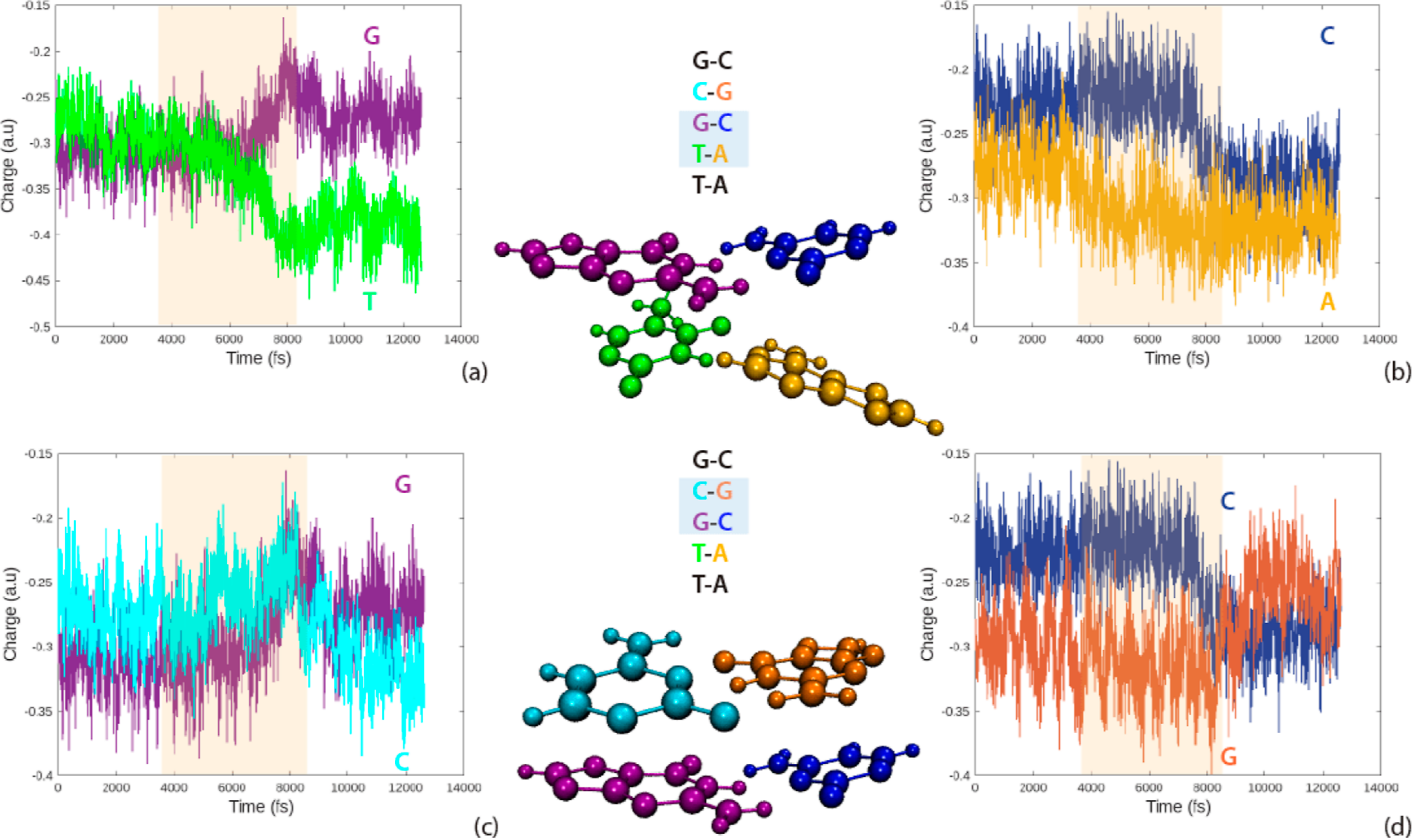
Mulliken charge variations of the individual nucleic acid bases in a stacking configuration for the sequence T–T–G–C–G. The shadowed regions indicate the time interval in the opening of the third base pair, GC. Snapshots of G, T (a) and their complementary bases C, A (b) in a stacking configuration and G, C (c) and their complementary bases C, G (d) in a stacking configuration are shown.

The three *DNA* sequences of this work have GC as the central base pair but different nucleic acid neighbors in the stacking configuration. Figure 8 reveals charge variations for the sequence T–T–G–C–G according to the stacking of the nucleic acid bases. Inset Figure 8a,b shows charge variations of GC and TA in tandem in the sequence T–T–G–C–G, and similarly, inset Figure 8c,d shows charge variations of GC and CG. In the process of strand separation, there is a small charge exchange between adjacent base pairs in the stacking conformation. This exchange ceases once the nucleic strands are separated. The electric charges of the nucleic acid bases for the other two sequences exhibit analogous behaviors.

### 3.5 Sequence-Dependent Forces and Energies

**Figure 9:**
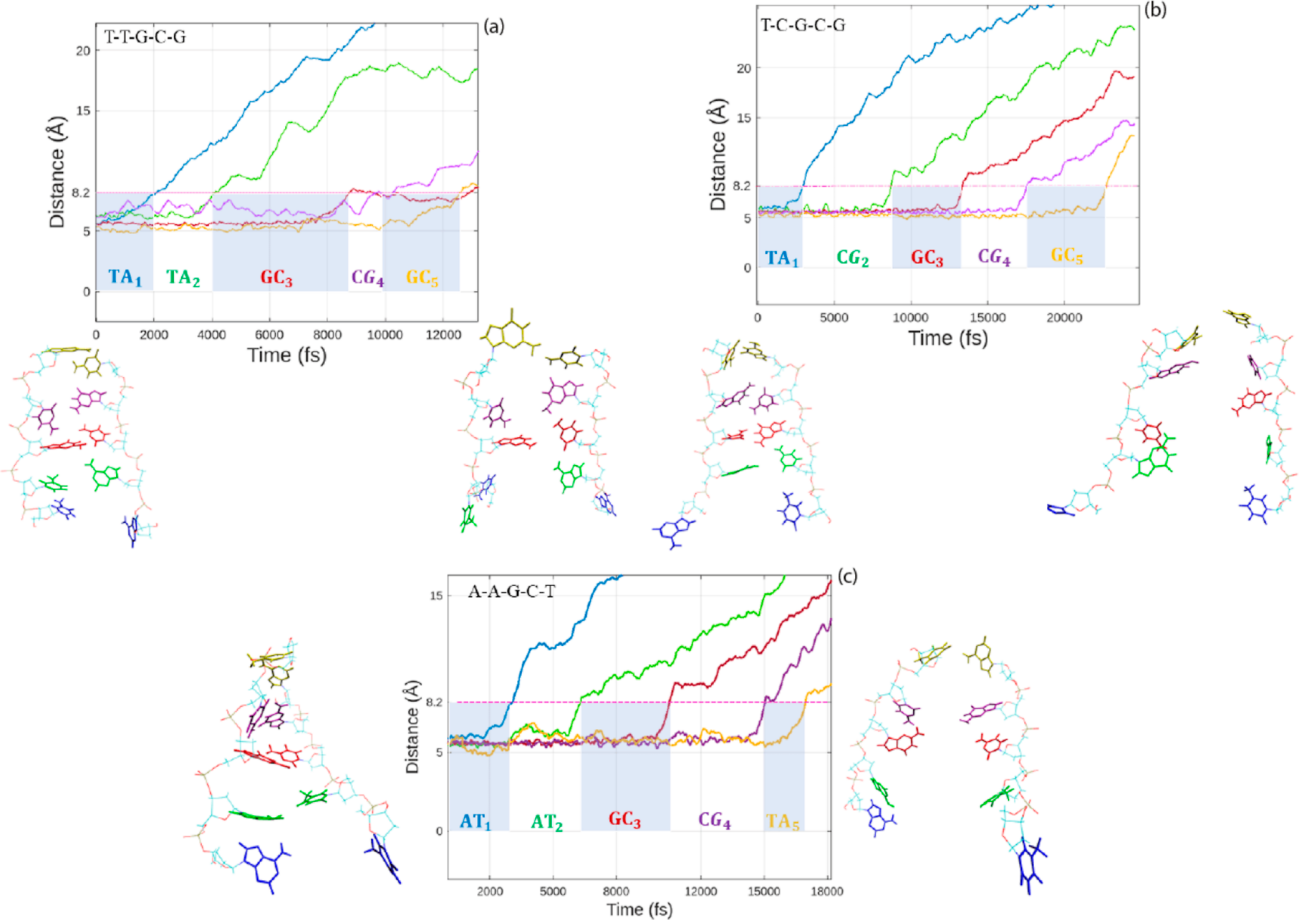
CM separation vs time. Refer to Figure 5 for the definition of the CM separation. The blue and white regions in succession indicate the time intervals of the harmonic force action on a base pair. Snapshots of the structure with 2 and 5 dissociated base pairs are shown for the different *DNA* sequences: T–T–G–C–G (a), T–C–G–C–G (b), and A–A–G–C–T (c), on the left- and right-hand sides, respectively.

To gain a better understanding of the underlying mechanisms of cellular processes, we further explored the separation of the nucleic strands in terms of their sequences. Figure 9 exhibits the sequential opening of the nucleic acid bases by plotting the separation of their CM with time for the *DNA* sequences T–T–G–C–G, T–C–G–C–G, and A–A–G–C–T.

The common central base pair in the structures is GC (the third base pair in each sequence). It is selected as a reference base pair to analyze sequence-dependent forces and energies. The central base pair rupture forces (with dispersion corrections in parentheses) are 922.022 (1113.990), 887.995 (1123.959), and 781.960 (1055.988) in pN units for sequences 1, 2, and 3, respectively. Taking into consideration that these forces were statistically computed (namely, with the existence of temperature fluctuations), such forces are relatively consistent with each other. Despite the statistical conditions, we note that for the *DNA* sequence with AT-rich composition, A–A–G–C–T, the force necessary to separate the central GC base pair remains the lowest when compared to that of the other sequences. This observation holds true regardless of whether dispersion corrections are taken into account or not.

The change in the average energies between the initial stage, prior to the application of harmonic forces, and the final stage of the simulation, where the *DNA* strands are completely separated and equilibrated, provides the net energies for the separation of the *DNA* sequences examined in this study. However, achieving equilibrium in this final stage of the system, when the two nucleic strands are separated and the springs are deactivated, presents considerable challenges. The two individual strands become constantly distorted, becoming difficult to equilibrate, even after simulations beyond 3 ps. The changes in the average energies (with dispersion corrections in parentheses) are 31.37 (29.55), 53.33 (11.295), and 75.363 (43.110) in kcal/mol units for sequences 1, 2, and 3, respectively. It is important to keep in mind that the findings of this work are obtained for spring forces acting under quasi-equilibrium conditions, which are similar to the way in which helicase works: the helicase applies a subtle force with the right alignment to smoothly open the *DNA* strands without tangling them or causing errors in the replication process.

## 4 Conclusions

Three different *DNA* sequences were analyzed to assess the mechanical behavior of *ds-DNA* in the instances of double-strand separation into two single strands due to the action of external harmonic forces. First-principles molecular dynamics were performed under the DFT scope. Accurate modeling of molecular systems using DFT requires careful evaluation of the dispersion corrections. Inconsistencies between dispersion-inclusive DFT methods and highly accurate reference calculations emphasize the importance of prior evaluation and testing of the dispersion corrections for accurate DFT calculations. In this regard, we present results at the B3LYP/6-31g* level of theory with and without the D3 dispersion correction term. The helicase-*DNA* interaction was modeled using a Langevin force approach. This methodology implicitly accounts for the presence of helicase and its impact on *DNA* strand separation, providing an accurate and computationally efficient representation of the environmental factors that affect the *DNA* helix. The separation of the *DNA* strands was achieved with harmonical forces applied in a sequential and helicoidal manner, simulating the helicase action as observed in cellular processes. One of the key features of molecular dynamics is its ability to account for time-dependent processes and derive statistics pertaining to energies, charges, thermal equilibration, and other related aspects. Recognizing that biological processes are inherently dynamic processes, our results include time-dependent graphs that showcase the dynamic aspects of the *DNA* double helix opening, together with the inherent statistical values obtained from those plots. By considering this approach, we obtained energy and temperature variations, dissociation forces, and electric charge fluctuations in the separation process. The magnitude of the forces typically takes hundreds of pN. In addition to the type of bases forming a Watson–Crick pair, the stacking configuration is an important factor that has to be also considered in the determination of force magnitudes. The forces with dispersion corrections are generally greater than those with no dispersion corrections, although the extent of this contribution varies with respect to the *DNA* sequence. In the separation moments of the double strand, when the system is out of equilibrium due to the external forces, charge fluctuations are observed. These fluctuations cease after the separation of the strands, achieving charge relaxation. The temperature changes are relatively small, while the energy is sensible to the sequence of the nucleic acid bases, exhibiting peaks in the separation of every base pair. The present model offers adaptability in the positioning and force intensity of springs, making it applicable in investigations of biological systems. As such, it serves as a valuable resource for conducting experiments using atomic force microscopes and optical tweezers in the context of *DNA* research.
